# The Death of Nicholas Senn

**Published:** 1908-02

**Authors:** 


### THE DEATH OF NICHOLAS SENN.

No physician in America was more extensively and more favorably known than was Nicholas Senn, who died Jan. 2nd, at his home in Chicago at the age of 63.

The life of Senn was a most inspiring and encouraging one, as he rose from the ranks of an humble practitioner, on account of his genuine greatness, to the foremost ranks of the leading surgeons of the world. He was born in Bucks, Switzerland, in the year 1844 and came to the United States in 1852. After two years practice in Ashford, Wisconsin, he moved to Milwaukee and later to Chicago. He was soon placed on the staff of the Cook County Hospital of the last named city, where his unusual ability was recognized and he soon had other honors heaped upon him. His first work as a teacher was as the professor of surgery in the College of Physicians and Surgeons of Chicago, and later he was made professor of this branch in the Rush Medical College. As a surgeon, Senn's reputation was world-wide, especially in plastic surgery. His name is also connected with numerous operative procedures which he devised.

He was well known in military circles, being on duty at Chicamauga Park during the Spanish-American war, and chief-surgeon of the operating staff in the field with the troops in Cuba.

Dr. Senn was a prolific writer of text books, as well as of contributions to the medical journals.

The glowing tribute to him in the Journal of the American Medical Association ends as follows:

"Nicholas Senn was truly great; master of his profession; a patriot, always ready to sacrifice his personal interests and comfort for the service of his adopted country; intensely loyal in his friendships; generous to a fault; simple minded; too honest to harbor suspicions; a man of singularly clean speech, never profane nor vulgar. His greatest glory was in his extraordinary capacity for work, which he held as duty, and that work entirely for the betterment of his fellow men. Of him it may with truth be said that the world is better for his having lived."

